# Association of the c-reactive protein-triglyceride glucose index with myocardial ischemia in patients with minimal to moderate coronary artery disease

**DOI:** 10.3389/fmed.2026.1904020

**Published:** 2026-08-20

**Authors:** Zengfa Huang, Beibei Cao, Xiaoyu Liu, Wen Shu, Wanpeng Wang, Mei Li, Zuoqin Li, Xi Wang, Jianwei Xiao, Xiang Li, Yun Hu, Xiang Wang

**Affiliations:** 1 Department of Radiology, The Central Hospital of Wuhan, Tongji Medical College, Huazhong University of Science and Technology, Wuhan, Hubei, China; 2 Department of Community Health, Hanyang District Center For Disease Control and Prevention, Wuhan, Hubei, China; 3 Department of Public Health, The Central Hospital of Wuhan, Tongji Medical College, Huazhong University of Science and Technology, Wuhan, Hubei, China

**Keywords:** coronary artery disease, coronary computed tomography angiography, c-reactive protein-triglyceride-glucose index, minimal to moderate coronary artery disease, myocardial ischemia

## Abstract

**Objective:**

This study sought to explore the relationship between the C-reactive protein-triglyceride-glucose index (CTI) and the incidence of myocardial ischemia in patients with minimal to moderate coronary artery disease (CAD).

**Methods:**

The research retrospectively included 707 patients who underwent coronary computed tomography angiography (CTA) and blood tests for triglycerides, glucose, and C-reactive protein (CRP). CTI was calculated as: CTI = 0.412 × Ln(CRP [mg/L]) + Ln(TG [mg/dl] × FPG [mg/dl])/2. The definition of myocardial ischemia included coronary computed tomography angiography-derived fractional flow reserve (CT-FFR) measurements at or below 0.80. Multivariable logistic regression and subgroup analysis were performed to assess the association between CTI and myocardial ischemia.

**Results:**

The mean age of these 707 patients with minimal to moderate CAD was 63.9 ± 10.2 years, with 52.9% being male. After adjusting for other basic characteristics and/or coronary CTA features, the highest CTI quartile (T4) was linked with an increased risk of myocardial ischemia across four models, with odds ratios (ORs) ranging from 2.11 to 2.59 (all *p* < 0.05). Moreover, increasing the CTI by 1-SD was associated with a 24–30% increased risk of myocardial ischemia in all four models (all *p* < 0.05). Subgroup analysis showed that segment involvement score (SIS) ≥ 2 and moderate stenosis (50%–69%) were positively correlated with myocardial ischemia and the CTI.

**Conclusion:**

The study demonstrates that CTI is independently associated with myocardial ischemia detected by CT-FFR in individuals with minimal to moderate CAD. Notably, the predictive strength of CTI is particularly significant in patients with higher plaque burdens (SIS ≥ 2) and moderate stenosis (50%–69%), suggesting its potential to further identify truly high-risk individuals within the minimal to moderate CAD population.

## Introduction

Coronary computed tomography angiography (CTA) is considered the best non-invasive approach for diagnosing coronary artery disease (CAD) (1). It is recommended as the initial imaging choice for patients experiencing chest pain according to clinical practice guidelines (2, 3). The primary focus of most studies has been on assessing and managing patients with obstructive CAD, which involves stenosis exceeding 70% or lumen stenosis greater than 50% (4). Nonetheless, earlier research indicates that a large percentage (as much as 70%) of patients undergoing coronary CTA have non-obstructive CAD (5). The detection of non-obstructive CAD via coronary CTA has been associated with a higher risk of cardiovascular events compared to patients without CAD (6, 7). Beyond this traditional definition, an even broader group of patients with minimal to moderate stenosis (1–69%) on CTA has recently gained increasing attention, as these patients represent a large and heterogeneous population in whom the risk of myocardial ischemia is often underestimated by anatomical assessment alone.

The rise and ongoing development of coronary computed tomography angiography-derived fractional flow reserve (CT-FFR) has significantly impacted the diagnosis and management strategies for CAD. CT-FFR has been recommended by recent consensus documents and guidelines for diagnosing, evaluating and guiding treatment decisions of CAD (3, 8–10).

Although CT-FFR enhances the ability to assess functional significance, biomarkers that integrate inflammation and insulin resistance still retain independent value. The triglyceride-glucose (TyG) index, which is based on fasting triglycerides and glucose, is a well-established marker for insulin resistance and is consistently related to cardiovascular disease risk and adverse health outcomes across various populations. The TyG index only captures the metabolic aspect of insulin resistance, neglecting the inflammatory component, which is also crucial in atherosclerosis and plaque instability. Given the synergistic effect of insulin resistance and vascular inflammation in promoting atherosclerosis, a marker that combines both pathways may offer a more detailed assessment of cardiovascular risk (11). The C-reactive protein-triglyceride-glucose index (CTI), developed by Ruan et al., is an innovative marker that integrates C-reactive protein (CRP) with the TyG index to simultaneously measure inflammation and insulin resistance (12). This dual-pathway method theoretically provides benefits over the TyG index alone, especially in groups where inflammation is a key factor, like those patients undergoing percutaneous coronary intervention (PCI) (13). In addition, emerging evidence establishes CTI as a robust and independent predictor of cardiovascular disease (CVD) (14–16). Its significant association with both CVD incidence and mortality has been consistently validated across diverse populations, notably including adolescents and individuals with metabolic disorders or diabetes (17–22). Unlike our previous work that focused solely on the TyG index (23), the present study investigates CTI, which integrates both insulin resistance and inflammation pathways.

However, studies examining the link between the CTI and the occurrence of myocardial ischemia in patients with minimal to moderate CAD are still missing. Thus, we sought to explore the relationship between the CTI and the incidence of myocardial ischemia in patients with minimal to moderate CAD.

## Methods

### Study population

**FIGURE 1:**
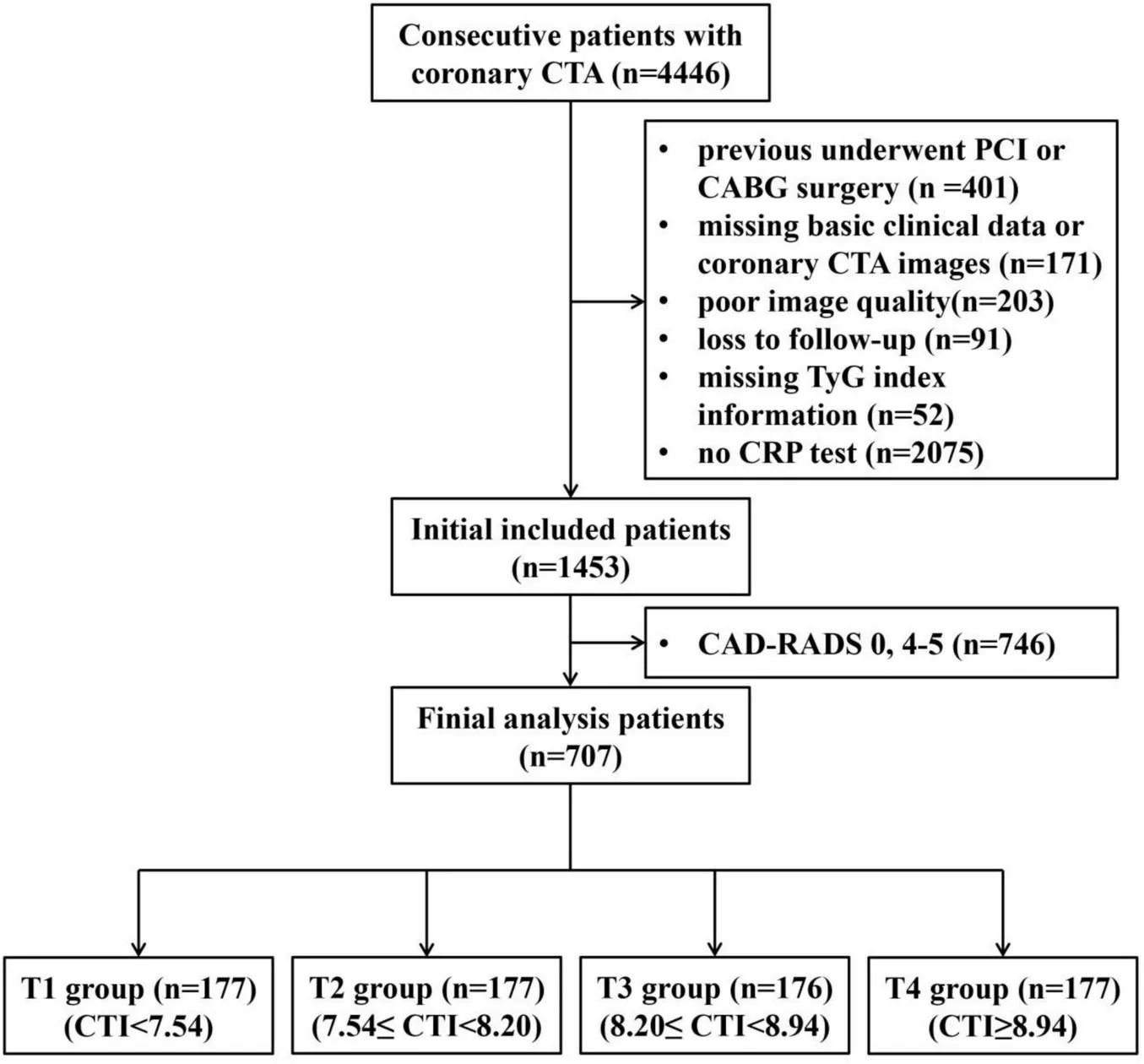
Flowchart of the study design. CTA, computed tomography angiography; PCI, percutaneous coronary intervention; CABG, coronary artery bypass grafting; TyG, triglyceride glucose; CRP, C-reactive protein; CAD-RADS, Coronary Artery Disease Reporting and Data System; CTI, C-reactive protein-triglyceride glucose index.

The research retrospectively included individuals who underwent coronary CTA testing from November 2018 to December 2020. Those eligible for inclusion were patients aged 18 or older who had blood tests for triglycerides, glucose, and CRP done within a week before the coronary CTA examination. Patients were excluded if they had undergone revascularization operations (coronary artery bypass grafting, CABG or, PCI), had a prior myocardial infarction (MI), were without coronary CTA images or reports, were without basic clinical data, were without triglycerides or glucose or CRP test, or were lost to follow-up. Minimal to moderate CAD was defined as 1%–69% diameter stenosis on coronary CTA. A total of 707 patients who qualified were eventually included in the study (Figure 1). The study protocol was approved by the Ethics Review Committee of The Central Hospital of Wuhan (WHZXKYKL2022-140) and the written informed consent was waived by the institutional ethics committee of The Central Hospital of Wuhan because of the retrospective observational nature of the study. We confirm that all methods were performed in accordance with the relevant guidelines and regulations.

### Data collection and definitions

Data on basic parameters (such as age, sex, smoking and drinking status), clinical history (like hypertension and diabetes), and laboratory test results were extracted from electronic medical records. The CTI, comprising CRP and TyG, was computed using the following formula:

CTI = 0.412 × ln (CRP [mg/L]) + ln (TG [mg/dl] × FPG [mg/dl])/2 (12).

### Coronary CTA acquisition and analysis

Coronary CTA scans were performed on a dual-source CT scanner (Somatom Definition Flash, Siemens Medical Solutions, Forchheim, Germany) with ECG triggering, which could be done prospectively or retrospectively. An AI tool (Computer Aided Diagnosis of Coronary Artery, Version 6.0, Shukun technology, Beijing, China) was employed for the automated examination of coronary CTA images, producing standardized post-processed images and structured reports. Previous research provided more in-depth information on this AI-driven coronary CTA platform (23, 24). According to the guidelines from the Society of Cardiovascular Computed Tomography, an 18-segment model was utilized to evaluate coronary anatomy. The stenosis of CAD severity was categorized into minimal or mild stenosis (1%–49%) and moderate stenosis (50%–69%). The segment involvement score (SIS) assessed plaque burden and was divided into SIS < 2 or SIS ≥ 2. The definition of myocardial ischemia included CT-FFR measurements at or below 0.80.

### Statistical analysis

Continuous variables that are not normally distributed were shown as medians with interquartile ranges (IQRs), while those that are normally distributed were presented as means with standard deviations (SDs). Categorical variables were expressed as absolute values with percentages. To compare continuous variables, one-way analysis of variance or the Kruskal–Wallis test was utilized. The comparison of categorical variables was conducted using Chi-square tests or Fisher’s exact tests. Univariable and multivariable logistic regression analyses, including odds ratios (ORs) and 95% confidence intervals (CIs), were used to examine the relationship between CTI and myocardial ischemia. Covariates were included in the multivariable models using a hybrid approach that combined statistical significance from univariable analyses (*P* < 0.05) with established clinical relevance from the literature. Age was additionally retained in all models despite a *P* > 0.05 in univariable analysis, given its well-recognized and clinically essential role as a cardiovascular risk factor. Four multivariable logistic regression models with different adjustment sets were constructed. Model 1 accounted for age, gender, smoking, drinking, hypertension, and diabetes. Model 2 included adjustments for moderate stenosis in addition to the factors in Model 1. Model 3 incorporated SIS adjustments, also based on Model 1. Model 4 included adjustments for both moderate stenosis and SIS, using Model 1 as a foundation. Additionally, the logistic regression results were displayed based on CTI quartiles (categorical variables), using the T1 group (lowest quartile) as the reference point, and by the increase per SD in the CTI (continuous variable) for interpretation of the results. The non-linear association between the CTI and myocardial ischemia was assessed using restricted cubic spline regression. Finally, analyses were stratified by sex, hypertension, SIS, and stenosis with adjustments made to the model. Statistical significance was determined at *P* < 0.05. Analyses were conducted using R statistical package (version 4.3.3, R foundation for Statistical Computing, Vienna, Austria) and SPSS (version 18, SPSS, Inc., Chicago, IL, USA).

## Results

### Basic characteristics of the study patient

In this study, 707 patients with minimal to moderate CAD were ultimately analyzed. The mean age of these patients was 63.9 ± 10.2 years, with 52.9% being male. The median CTI was 8.2, with an interquartile range of 7.54 to 8.94. Patients in the T4 group tended to be younger and had a history of diabetes compared to those in the T1 group. No differences were observed in the distribution of other basic characteristics and coronary CTA features across the groups (Table 1). Baseline characteristics stratified by the presence or absence of myocardial ischemia (CT-FFR ≤ 0.80 vs. > 0.80) are presented in Supplementary Table 1.

**TABLE 1:** Basic characteristics of the study patients.

Variables	Total patients (*n* = 707)	CTI (in quartiles)				*P* value
		T1 group (*n* = 177, CTI < 7.54)	T2 group (*n* = 177, 7.54 ≤ CTI ≤ 8.20)	T3 group (*n* = 176, 8.20 ≤ CTI ≤ 8.94)	T4 group (*n* = 177, CTI > 8.94)	
Age (years)	63.9 ± 10.2	65.8 ± 9.4	64.2 ± 9.4	63.8 ± 9.8	61.7 ± 11.8	0.002
Gender (Male, n, %)	374 (52.9)	106 (59.9)	90 (50.8)	91 (51.7)	87 (49.2)	0.182
Smoke	187 (26.4)	44 (24.9)	47 (26.6)	49 (27.8)	47 (26.6)	0.939
Drink	101 (14.3)	19 (10.7)	31 (17.5)	25 (14.2)	26 (14.7)	0.340
Hypertension	370 (52.3)	84 (47.5)	87 (49.2)	101 (57.4)	98 (55.4)	0.182
Diabetes	149 (21.1)	29 (16.4)	32 (18.1)	34 (19.3)	54 (30.5)	0.004
Moderate Stenosis (50%–69%)	236 (33.4)	54 (30.5)	56 (31.6)	69 (39.2)	57 (32.2)	0.297
SIS ≥ 2	163 (23.1)	37 (20.9)	39 (22.0)	41 (23.3)	46 (26.0)	0.698
CT-FFR ≤ 0.8	128 (18.1)	24 (13.6)	29 (16.4)	33 (18.8)	42 (23.7)	0.084

CTI, C-reactive protein-triglyceride-glucose index; SIS, Segment Involvement Score; CT-FFR, CT derived-fractional flow reserve.

### Association between CTI and myocardial ischemia

**FIGURE 2:**
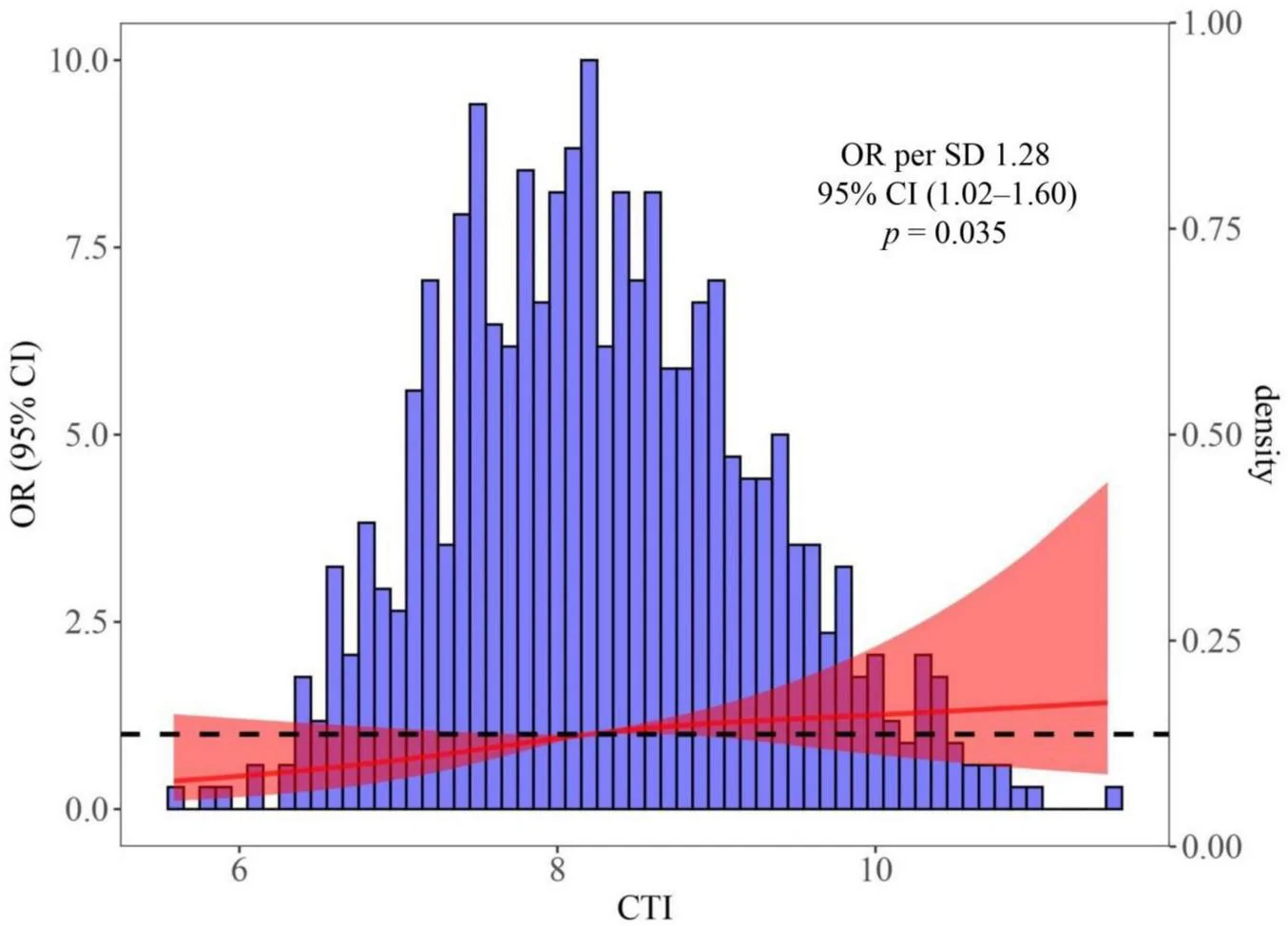
The restricted cubic spline of CTI and the incidence of myocardial ischemia. CTI, C-reactive protein-triglyceride glucose index; OR, odds ratio; CI, confidence interval; SD, standard deviation.

To explore the non-linear link between the CTI and myocardial ischemia, flexible modeling was conducted using restricted cubic splines. The curve showed that the OR (per SD) for the development of myocardial ischemia was 1.28 (95%CI, 1.02–1.60, *p* = 0.035) (Figure 2).

**FIGURE 3:**
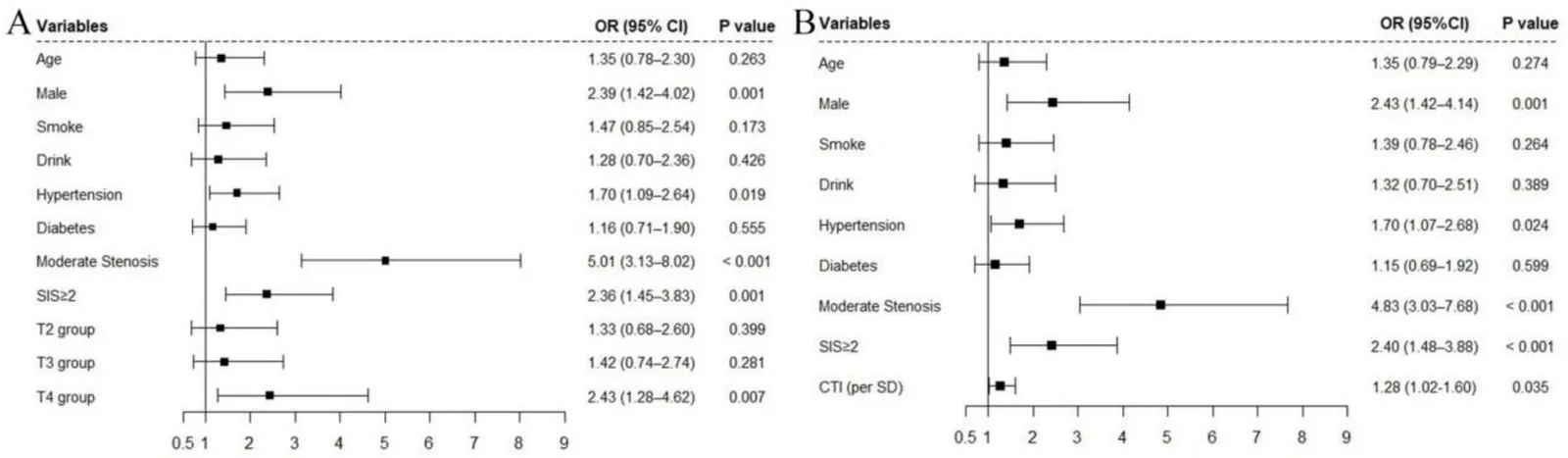
Risk of myocardial ischemia based on model 4. CTI was regarded as categorical variable **(A)** and continuous variable **(B)**. Model 4 was adjust by age, gender, smoke, drink, hypertension, diabetes, moderate stenosis, SIS. CTI, C-reactive protein-triglyceride glucose index; SIS, segment Involvement Score.

Univariable analysis showed that the T4 group had a significantly higher likelihood of myocardial ischemia than the T1 group (OR, 1.98; 95% CI, 1.14–3.45; *p* = 0.015). When CTI was analyzed as a continuous variable, the outcome per SD was consistent (OR, 1.24; 95% CI, 1.02–1.51; *p* = 0.029) (Table 2). Even after accounting for other basic characteristics and/or coronary CTA features, the T4 group remained significantly associated with an increased risk of myocardial ischemia in Models 1, 2, 3, and 4 (OR, 2.21, 95% CI, 1.23–3.98, *p* = 0.008; OR, 2.59, 95% CI, 1.38–4.89, *p* = 0.003; OR, 2.11, 95% CI, 1.15–3.89, *p* = 0.016; OR, 2.43, 95% CI, 1.28–4.62, *p* = 0.007) (Figure 3A; Table 3). Moreover, increasing the CTI by 1-SD was associated with a 24-30% increased risk of myocardial ischemia in all four models (all *p* < 0.05) (Figure 3B; Table 3). Among the covariates included in the multivariable models, male sex and hypertension were also significantly associated with an increased risk of myocardial ischemia (Figures 3A, B). Due to the retrospective nature of this study and incomplete data capture in the electronic medical records, several potential confounders – including body mass index (BMI), dyslipidemia, family history of CAD, and detailed medication use (e.g., statins, antidiabetic, and anti-inflammatory therapies) – could not be included in the multivariable models because of substantial missing data.

**TABLE 2:** Univariate logistic regression analysis for myocardial ischemia

Variables	Univariate	
	OR (95% CI)	*P* value
Age (≥60 years)	1.34 (0.86–2.09)	0.189
Gender (Male)	2.61 (1.77–4.09)	<0.001
Smoke	2.15 (1.44–3.23)	<0.001
Drink	2.19 (1.36–3.53)	0.001
Hypertension	2.13 (1.42–3.18)	<0.001
Diabetes	1.61 (1.04–2.50)	0.032
Moderate Stenosis (50%–69%)	6.71 (4.41–10.21)	<0.001
SIS ≥ 2	4.85 (3.22–7.30)	<0.001
CTI (quartiles)		
T2 Group	1.25 (0.70–2.25)	0.457
T3 Group	1.47 (0.83–2.61)	0.187
T4 group	1.98 (1.14–3.45)	0.015
CTI (Per SD)	1.24 (1.02–1.51)	0.029

CTI, C-reactive protein-triglyceride-glucose index; SD, standard deviation; SIS, Segment Involvement Score; CT-FFR, CT derived-fractional flow reserve; OR, odds ratios.

**TABLE 3:** Association between CTI and myocardial ischemia.

Characteristics	Model 1		Model 2		Model 3		Model 4	
	OR (95% CI)	*P* value	OR (95% CI)	*P* value	OR (95% CI)	*P* value	OR (95% CI)	*P* value
CTI (per SD)	1.26 (1.03–1.54)	0.028	1.30 (1.04–1.62)	0.023	1.24 (1.00–1.54)	0.047	1.28 (1.02–1.60)	0.035
CTI (quartiles)								
T1 group	1 reference		1 reference		1 reference		1 reference	
T2 group	1.35 (0.73–2.49)	0.338	1.39 (0.72–2.70)	0.326	1.31 (0.69–2.47)	0.405	1.33 (0.68–2.60)	0.399
T3 group	1.58 (0.87–2.89)	0.137	1.47 (0.77–2.80)	0.239	1.48 (0.80–2.78)	0.215	1.42 (0.74–2.74)	0.281
T4 group	2.21 (1.23–3.98)	0.008	2.59 (1.38–4.89)	0.003	2.11 (1.15–3.89)	0.016	2.43 (1.28–4.62)	0.007
*P* value for trend		0.060		0.023		0.103		0.042

CTI, C-reactive protein-triglyceride-glucose index; SD, standard deviation; SIS, Segment Involvement Score; CT-FFR, CT derived-fractional flow reserve; CI, confidence intervals; OR, odds ratios. Model 1, adjust by age, gender, smoke, drink, hypertension, diabetes; Model 2, adjust by age, gender, smoke, drink, hypertension, diabetes, moderate stenosis; Model 3, adjust by age, gender, smoke, drink, hypertension, diabetes, SIS; Model 4, adjust by age, gender, smoke, drink, hypertension, diabetes, moderate stenosis, SIS.

### Subgroup analysis

**FIGURE 4:**
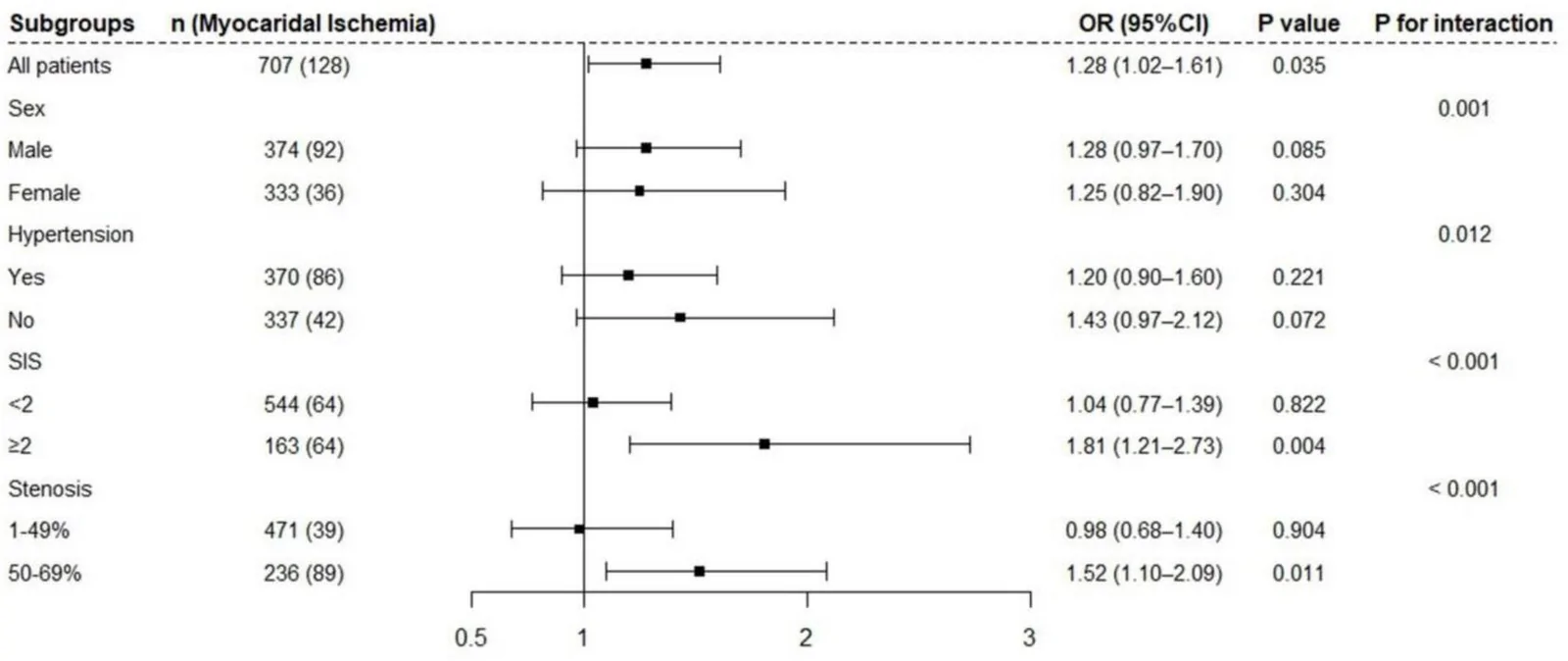
Stratified analyses for the association of CTI with myocardial ischemia based on model 4. Model 4 was adjusted by adjust by age, gender, smoke, drink, hypertension, diabetes, moderate stenosis, SIS. CTI, C-reactive protein-triglyceride glucose index; SIS, segment Involvement Score.

The relationships between the CTI and myocardial ischemia were further assessed across various subgroups by accounting for confounding factors. Subgroup analysis showed that SIS ≥ 2 and moderate stenosis (50%-69%) were positively correlated with myocardial ischemia and the CTI. Additionally, being male and not having hypertension showed a borderline positive correlation with myocardial ischemia and CTI (Figure 4).

## Discussion

To our knowledge, this study is the first to demonstrate that the novel inflammation-insulin resistance composite index (CTI) is independently linked to myocardial ischemia occurrence, as detected by CT-FFR, in patients with minimal to moderate CAD. Even after fully adjusting for traditional risk factors and coronary CTA features, CTI still significantly predicted the risk of myocardial ischemia. In addition, subgroup analysis indicated that the relationship between CTI and myocardial ischemia was particularly evident in patients with SIS ≥ 2 and those with moderate stenosis (50%–69%), highlighting its enhanced role for risk stratification in these higher-risk subgroups.

Studies conducted previously have demonstrated that CTI can act as a valuable tool for predicting CVD outcomes. For example, Sun and colleagues examined 8,679 adults from the National Health and Nutrition Examination Survey (NHANES) and found a significant link between increased CTI and the incidence and mortality of CVD (17). Xu et al. validated its effectiveness in identifying coronary heart disease prevalence in the NHANES general population (20). A recent study involving 5,642 participants aged 45 and older from the China Health and Retirement Longitudinal Study (CHARLS) found a significant association between higher CTI and an increased risk of CVD (16). Furthermore, numerous recent studies have shown a link between CTI and both all-cause and cardiovascular mortality (17, 25–27). Unlike earlier research that concentrated on the general population, this study shifts its focus to patients with minimal to moderate CAD (1–69% stenosis), a population that accounts for a substantial proportion yet remains understudied, and uses CT-FFR, a functional marker, as the result metric instead of just focusing on anatomical stenosis. This method confirms and broadens the insights about CTI in this specific population.

Recently, the TyG index has also been shown to predict adverse outcomes in various high-risk CAD subsets. For instance, Söner et al. demonstrated that the TyG index was an independent predictor of contrast-induced nephropathy (CIN) in patients with chronic total occlusion (CTO) undergoing PCI, with an odds ratio of 2.17 (95% CI: 1.21–3.89, *P* = 0.009) after multivariable adjustment (28). The study emphasizes the importance of insulin-resistance indices for prognosis, surpassing traditional risk factors. Although the TyG index measures the metabolic component of insulin resistance, it does not include systemic inflammation, another important element in the development of atherosclerosis, plaque instability, and myocardial infarction. Alternatively, the CTI unites CRP with the TyG index, thereby concurrently addressing both insulin resistance and inflammation pathways. This dual-pathway strategy might provide additional prognostic benefits beyond TyG alone (13), especially in groups where inflammation is a key factor, like patients with minimal to moderate CAD or those with a significant plaque burden. Our findings validate this hypothesis, as the correlation between CTI and CT-FFR-defined ischemia continued to be strong even after making extensive adjustments for traditional risk factors and coronary anatomical aspects. While the TyG index has proven valuable in predicting procedural complications like CIN in high-risk anatomical subsets (e.g., CTO), CTI may provide a more comprehensive risk assessment by integrating the inflammatory burden, making it particularly suitable for the broader and more heterogeneous population of patients with minimal to moderate CAD.

The potential mechanisms underlying this association may be explained the fact that CTI combines the TyG index, indicative of insulin resistance, with CRP, a marker of systemic inflammation, possibly providing a more thorough representation of the pathological foundation of atherosclerosis. For example, insulin resistance may cause endothelial dysfunction and issues with lipid metabolism, while the low-grade systemic inflammation indicated by CRP encourages the formation, development, and instability of plaques (29–32). An interaction effect between CTI and both SIS ≥ 2 and moderate stenosis related to myocardial ischemia was revealed by the subgroup analysis in this study. This could be due to the fact that myocardial ischemia can still be triggered by inflammation and insulin resistance, even without significant luminal stenosis, through mechanisms like endothelial dysfunction, microvascular disease, vasospasm, or plaque progression (33–36).

The outcomes of this study could have distinct clinical implications. CTI is a composite index formed from standard laboratory parameters (like lipids, glucose, and CRP), making it straightforward to calculate, cost-efficient, and easily applicable in clinical practice. Among the large and historically underexplored population of patients with minimal to moderate CAD, CTI effectively identifies those at higher risk for myocardial ischemia, complementing the limitations of relying solely on anatomical assessment. Moreover, subgroup analyses demonstrated that the relationship between CTI and myocardial ischemia was particularly evident in patients with SIS ≥ 2 and those with moderate stenosis (50%–69%). This observation suggests that CTI may aid in more precise risk categorization for patients with elevated plaque levels or borderline stenosis, helping to identify those who actually need enhanced treatment strategies. In addition, with the rise in availability of functional assessment techniques like CT-FFR, merging these anatomical and functional assessments with CTI may allow for a multidimensional ‘anatomical-functional-biological’ approach to cardiovascular risk assessment. This combined approach offers clinicians fresh perspectives for customizing personalized treatment plans, including more intensive anti-inflammatory therapies or drug interventions aimed at insulin resistance. Thus, CTI serves as a straightforward and efficient method for risk stratification, offering considerable promise for clinical application in managing patients with minimal to moderate CAD.

The current study also has several limitations. First, due to its retrospective and observational single-center design, there is a risk of patient selection bias, and causality cannot be inferred. Second, although there were 707 patients in total, the sample sizes for specific subgroups in the analyses were relatively limited. Notably, the correlation between CTI and myocardial ischemia in male patients and those without hypertension was only marginally statistically significant, which could be attributed to insufficient statistical power. Therefore, these results should be interpreted carefully and need to be confirmed in larger prospective studies. Third, this study utilized CT-FFR as the indicator for myocardial ischemia. Although it has undergone extensive validation, its accuracy might be compromised by factors like coronary calcification and image quality, and it may not be completely equivalent to invasive FFR. In addition, coronary slow flow phenomenon – which is not detectable by CTA – represents another potential mechanism of ischemia in patients without significant luminal obstruction. This further underscores the multifactorial nature of ischemia in minimal to moderate CAD and the potential role of inflammatory and metabolic factors in modulating microvascular function. Fourth, we did not exclude patients receiving statins, antidiabetic agents, or anti-inflammatory therapies. Because detailed medication records – including drug types, dosages, and treatment durations – were not systematically available in our retrospective study; we could not fully assess the potential impact of these medications on CTI-related parameters (CRP, triglycerides, and glucose). This may have introduced residual confounding. Fifth, CTI was derived from a one-time measurement of blood biomarkers, which might not reflect the evolving changes in inflammation and insulin resistance over time and their prolonged effect on the risk of myocardial ischemia. Sixth, even after accounting for several conventional cardiovascular risk factors, there may still be confounding effects from unmeasured variables lifestyle factors, other inflammatory biomarkers, BMI, dyslipidemia, and family history of CAD. Finally, the single-center nature and the lack of an external validation cohort limit the generalizability of our findings to different populations and healthcare settings. Further confirmation of our findings requires future multicenter, prospective studies with external validation cohorts.

## Conclusion

In conclusion, the study demonstrates that CTI, a novel composite index of inflammation and insulin resistance, independently associated with myocardial ischemia detected by CT-FFR in individuals with minimal to moderate CAD. Originating from routine lab parameters, this practical biomarker merges insights into inflammatory and insulin resistance pathways, serving as a valuable supplement to existing risk stratification strategies focused solely on anatomical assessment. Notably, the predictive strength of CTI is particularly significant in patients with higher plaque burdens (SIS ≥ 2) and moderate stenosis (50%–69%), suggesting its potential to further identify truly high-risk individuals within the minimal to moderate CAD. These insights provide a new framework for the early detection of myocardial ischemia risk and the development of personalized intervention plans. Further multicenter prospective studies with external validation groups are needed to verify our results and aid in their application to clinical practice.

## Data Availability

The original contributions presented in the study are included in the article/Supplementary material, further inquiries can be directed to the corresponding authors.
