# Supplementary material for: Association of the c-reactive protein-triglyceride glucose index with myocardial ischemia in patients with minimal to moderate coronary artery disease
Source: Front Med (Lausanne). 2026 Aug 20;13:1904020. doi: 10.3389/fmed.2026.1904020 (PMC13538120; doi:10.3389/fmed.2026.1904020)
Supplement: Supplementary file 1 [file Table_1.docx]

| **Supplementary Table S1. Baseline characteristics stratified by the presence of myocardial ischemia.** | | | |  |
| --- | --- | --- | --- | --- |
| **Variables** | **Variables** | **Ischemia (CT-FFR ≤ 0.80) (n=128)** | **Non-ischemia (CT-FFR > 0.80) (n=579)** | **P Value** |
| Age (years) | 63.9 ± 10.2 | 64.7 ± 10.4 | 63.7 ± 10.2 | 0.422 |
| Gender (Male, n, %) | 374 (52.9) | 92 (71.9) | 282 (48.7) | < 0.001 |
| Smoke | 187 (26.4) | 51 (39.8) | 136 (23.5) | < 0.001 |
| Drink | 101 (14.3) | 30 (23.4) | 71 (12.3) | 0.001 |
| Hypertension | 370 (52.3) | 86 (67.2) | 284 (49.1) | < 0.001 |
| Diabetes | 149 (21.1) | 36 (28.1) | 113 (19.5) | 0.031 |
| Moderate Stenosis (50%-69%) | 236 (33.4) | 89 (69.5) | 147 (25.4) | < 0.001 |
| SIS ≥2 | 163 (23.1) | 64 (50) | 99 (17.1) | < 0.001 |
| CTI | 8.3 ± 1.0 | 8.4 ± 1.0 | 8.2 ± 1.0 | 0.028 |

CTI, C-reactive protein-triglyceride-glucose index; SIS, Segment Involvement Score; CT-FFR, CT derived-fractional flow reserve.
